# Hepatitis B virus genotypes A1 and A2 have distinct replication phenotypes due to polymorphisms in the HBx gene

**DOI:** 10.1371/journal.ppat.1012803

**Published:** 2025-01-09

**Authors:** Min Zhang, Karim Mouzannar, Zhensheng Zhang, Yuji Teraoka, Jason Piotrowski, Yuji Ishida, Chise Tateno-Mukaidani, Takeshi Saito, Hiromi Abe-Chayama, Kazuaki Chayama, T. Jake Liang

**Affiliations:** 1 Liver Diseases Branch, NIDDK, NIH, Bethesda, Maryland, United States of America; 2 Department of Gastroenterology and Metabolism, Graduate School of Biomedical & Health Science, Hiroshima University, Hiroshima, Japan; 3 Division of Gastrointestinal and Liver Diseases, Department of Medicine, University of Southern California, Keck School of Medicine, Los Angeles, California, United States of America; 4 PhoenixBio Co., Ltd., Higashi-Hiroshima, Hiroshima, Japan; 5 Center for Medical Specialist Graduate Education and Research, Hiroshima, Japan; 6 Collaborative Research Laboratory of Medical Innovation, Hiroshima University, Hiroshima, Japan; Pennsylvania State University College of Medicine: Penn State College of Medicine, UNITED STATES OF AMERICA

## Abstract

HBV genotype A has two major subtypes, A1 (commonly in Africa) and A2 (commonly in Europe) with only 4% nucleotide differences. Individuals infected with these two subtypes appear to have different clinical manifestations and virologic features. Whether such a difference results from the virus or host has not been established. Using HBV generated from molecule clones of subtypes A1 and A2 in cell culture (HBVcc), we demonstrate that HBVcc of subtypes A1 and A2 can be passaged *in vitro* and *in vivo* and respond equally well to human IFN-α treatment. HBVcc passaged in human liver chimeric mice (HBVmp) infected human hepatocytes more efficiently than that of the original HBVcc. Subtype A2 showed a much higher viral replication level than that of subtype A1. Mechanistic investigations using constructs with chimeric A1/A2 sequences and specific mutations indicated that subtype A2 has an inherently higher replication phenotype due to specific polymorphisms in the *HBx* gene resulting in amino acid variations. Studies of HBx expression demonstrated that A1 HBx is expressed at a much lower level than that of A2 HBx. Mutagenesis studies identified two HBx amino acid variations responsible for the observed phenotypic difference. Using AlphaFold2, we generated structural models of HBx proteins of A1 and A2. Superposition of the two models reveal that the overall structural motifs are similarly aligned, except for the C-terminal peptides diverging between the A1 and A2 models, possibly explaining their functional difference. In conclusion, using various *in vitro* and *in vivo* models, here we show that subtype A2 has an inherently higher replication phenotype due to polymorphisms in HBx that result in possible differences in structure and expression level of the two subtype HBx proteins. This genotypic difference potentially explains the reported clinical differences between the two subtypes as well as providing a previously unrecognized association between viral sequence variations and clinical manifestations of HBV infection in humans.

## Author summary

HBV is a leading cause of liver disease worldwide. HBV genotypes have distinct geographical distributions, routes of transmission, virological features and clinical characteristics. HBV subtypes A1 and A2, despite being classified within one genotype, appear to have distinct virologic and clinical features. Whether such differences among the diverse HBV genotypes resulting from the virus or host has not been established mostly because of limitations in cell culture tools and models. Here we establish and apply infectious *in vitro* and *in vivo* systems to study the virological behaviors and treatment responses of HBV A1 and A2. The surprising and intriguing finding of subtype A2 having a higher replication phenotype because of polymorphisms in the *HBx* gene provides the first indication that sequence variations, specifically in *HBx* gene here and possibly elsewhere on the genome, may underpin the diverse clinical phenotypes of HBV infections. This study underscores the value of this platform in investigating HBV biology and the need to study genotype-phenotype differences among the diverse HBV genotypes that may offer unique insight into functions of viral proteins and regulatory elements.

## Introduction

Currently, 3.5% of the global population is chronically infected with HBV [1,2]. The distribution of chronic HBV infection varies substantially in the world. The prevalence of chronic HBV infection is higher in the Western Pacific (6.2% or 115 million individuals have a chronic HBV infection) and African regions (6.1% or 60 million individuals) [1,3]. Vaccination remains the most effective tool to prevent HBV infection. There has been a steady decline of prevalence of chronic HBV infection overall in the past 30 years [1,4,5]. However, despite the advent of effective vaccines, as well as other disease control measures like antiviral therapy, HBV infection is still a serious global health problem [1].

HBV consists of 9 major genotypes (A to I), one minor genotype (designated J) and multiple sub-genotypes (subtypes) based on divergence of the entire genomic sequence of >7.5% and 4–7.5%, respectively [6,7]. HBV subtypes A1 and A2, despite having less sequence variations, have distinct geographical distributions, routes of transmission and virological features. Subtype A1 (originally names as Aa) is found in Africa, whilst A2 (Ae) in Europe and North America. Subtype A1 isolates have greater mean nucleotide (nt) divergences than those of A2 and cluster on a phylogenetic branch separate from that of subtype A2 [8,9]. This finding suggests that subtype A1 has been endemic and a long natural history within these populations [10,11]. Subtype A1 transmission is perinatal, whilst A2 is mainly acquired by adults parenterally or sexually. Rapid progression to liver disease and hepatocellular carcinoma (HCC) has been linked to A1 [6].

Subtype A1, which differs from A2 by 4% at the nucleotide level, has been implicated in rapid progression to HCC in young black African males without cirrhosis [10,12–14]. Signature mutations in the basal core promoter (BCP) and pre-core sequence, in addition to the canonical A1762T/G1764A BCP mutations associated with reduced HBeAg expression and early seroconversion, have been described for the subtype A1 [10,12,15,16]. Case-controlled studies have shown that patients infected with the A1 have lower HBV DNA levels in both HBeAg-positive and -negative phases of chronic disease compared with those of A2 and D [15].

Tools and models to investigate HBV genotypes have been limited and few studies explore the differences and characteristics of various genotypes *in vitro* and *in vivo*. We previously described the production of various infectious HBV genotypes and studied their virologic behaviors, infection courses and treatment responses in cell culture and human liver chimeric mouse model [17]. Here we extended our study to compare the infection courses, virological features and treatment responses of HBV subtypes A1 and A2 and revealed interesting phenotypic differences between the two closely related subtypes. We also showed that variations in the *HBx* gene is responsible for the increased replication phenotype of A2.

## Results

### Establishment of cell line producing HBVcc of subtype A1 or A2

After transfection of HBV genomes of subtype A1 or A2 into HepG2 cells and selection with hygromycin B, we obtained a total of 192 clones of subtype A1 with 14 ones positive for HBeAg production in the culture supernatant and 180 clones of subtype A2 with 28 ones positive for HBeAg (generation of subtype A2 clones had been described previously) [17]. For those clones with positive HBeAg expression, we analyzed HBV DNA levels in the supernatant and selected one clone per subtype with the highest level of HBV DNA as our candidate stable cell lines (S1 Fig). We then expanded these clones and collected and concentrated the supernatant as stock of HBVcc for subsequent infection experiment.

### HBVcc subtypes A1 and A2 infect PXB-cells and human liver chimeric mice with a higher replication phenotype of A2

**Fig 1:**
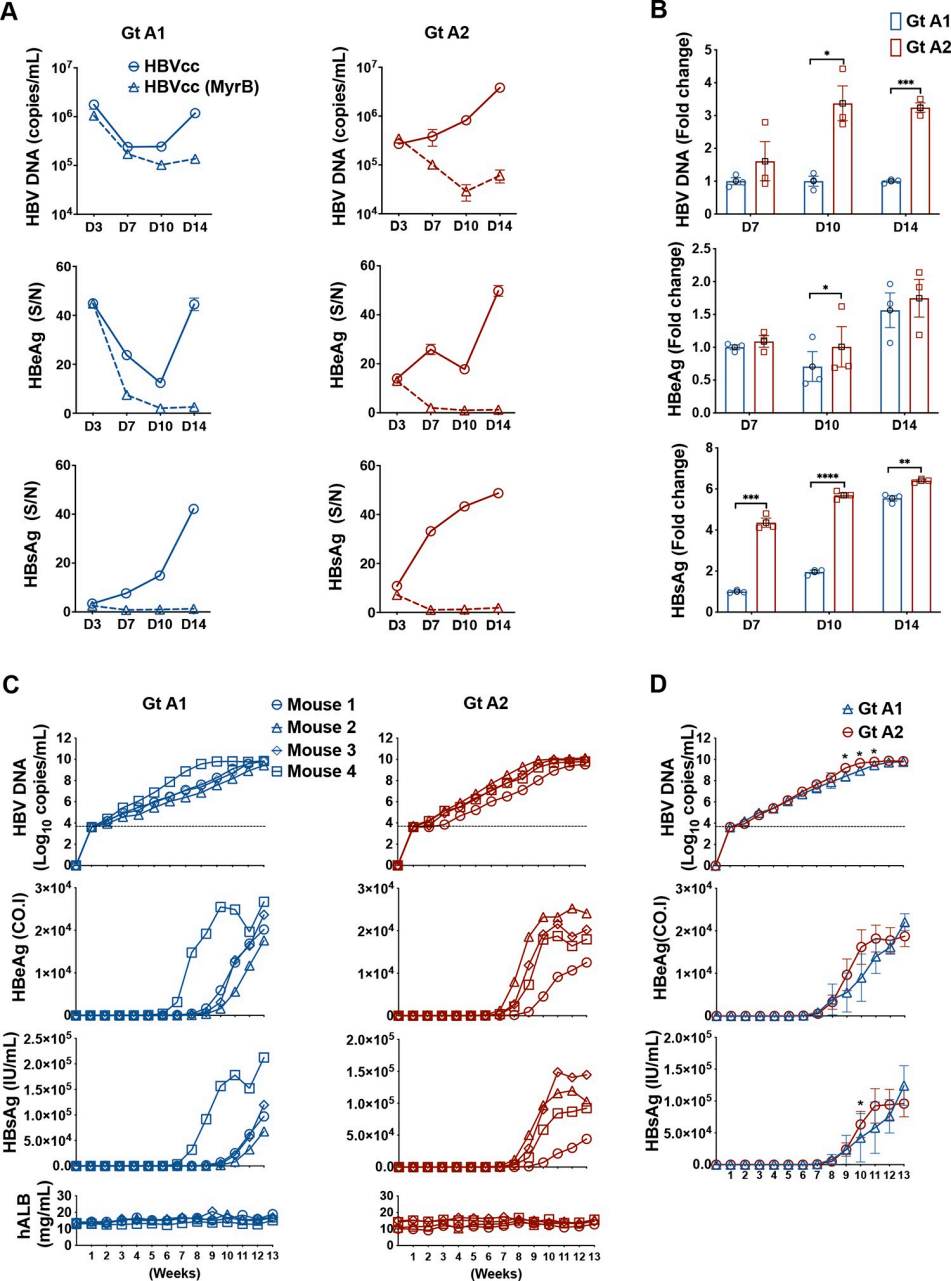
Infection of PXB-cells and human liver chimeric mice with HBVcc subtypes A1 and A2. **(A)** The infection course of subtypes A1 and A2 at different time points. PXB-cells were infected with HBVcc of subtypes A1 and A2 at an MOI of 10 (open circles with solid line). Another set of PXB-cells were infected in the presence of Myrcludex B (open triangles with dotted line). HBV DNA was performed with real-time qPCR and HBeAg /HBsAg were tested with ELISA at days 3, 7, 10 and 14 post-infection. The data from the ELISA is calculated as signal to noise ratio (S/N), with N defined as the average OD reading of negative control. The ELISA is generally linear up to a S/N of 50. **(B)** Comparison of HBV DNA, HBeAg and HBsAg between the two subtypes at different time points. Data are shown as mean ± SEM of triplicates. **** *P*<0.0001; *** *P*<0.001; ** *P*<0.01; * *P*<0.05. **(C)** The infection course of subtypes A1 and A2 in 4 mice per group for 13 weeks. Human liver chimeric mice were infected intravenously through tail vein injection with 1×10^6^ copies of HBVcc subtype A1 or A2. Serum was harvested weekly. The data of each mouse are denoted by different symbols. **(D)** Comparison of HBV DNA, HBeAg and HBsAg between the two subtypes at different time points. Data are shown as mean (in black) ± SEM of quadruplicates. Statistical analysis was performed with unpaired multiple *t* test after normalization accordingly (HBV DNA was normalized to week 2, HBeAg was normalized to week 6, and HBsAg was normalized to week 5 to compare their fold changes). **** *P*<0.0001; *** *P*<0.001; ** *P*<0.01; * *P*<0.05.

To examine the infectivity of virus produced by these stable cell clones, we concentrated and purified the HBVcc with heparin column and then infected PXB-cells (human hepatocytes passaged and expanded in human liver chimeric mice) at a multiplicity of infection (MOI) of 10. We collected the supernatant at different time points to monitor the expression of HBV DNA, HBeAg, and HBsAg (from days 3 to 14 post-infection). As shown in Fig 1A, both subtypes exhibited robust infection with a gradual increase of all the viral markers. The apparently higher HBV DNA levels at day 3 post-infection can probably be attributed to HBV input. As expected, the production of viral markers was blocked by Myrcludex B at the time of infection (dotted lines). Interestingly, subtype A2 produced significantly higher levels of HBV DNA and HBsAg (higher HBeAg but not significant) comparing to A1 at different time points (Fig 1B).

To compare the replication of A1 and A2 *in vivo*, human liver chimeric mice (n = 4 per subtype) were inoculated with a titer of 1x10^6^ copies of each subtype and monitored for 26 weeks. Weekly blood samples were obtained and measured for human albumin (hAlb), HBsAg, HBeAg and HBV DNA. As shown in Fig 1C, all inoculated mice demonstrated gradual increases of serum HBV DNA, HBeAg, and HBsAg. The hAlb levels were stable in all mice during the experimental period, indicating the maintenance of the engrafted human liver cells. In accordance with what we found in PXB-cells infection, subtype A2 showed a trend of higher HBV markers in early stage of infection process (before week 12). Even with a small number of mice, there were statistical differences at week 9 to 11 for HBV DNA level and week 10 for HBsAg expression (Fig 1D).

### HBV subtypes A1 and A2 respond similarly to hIFN-α2a in PXB-cells and human liver chimeric mice

**Fig 2:**
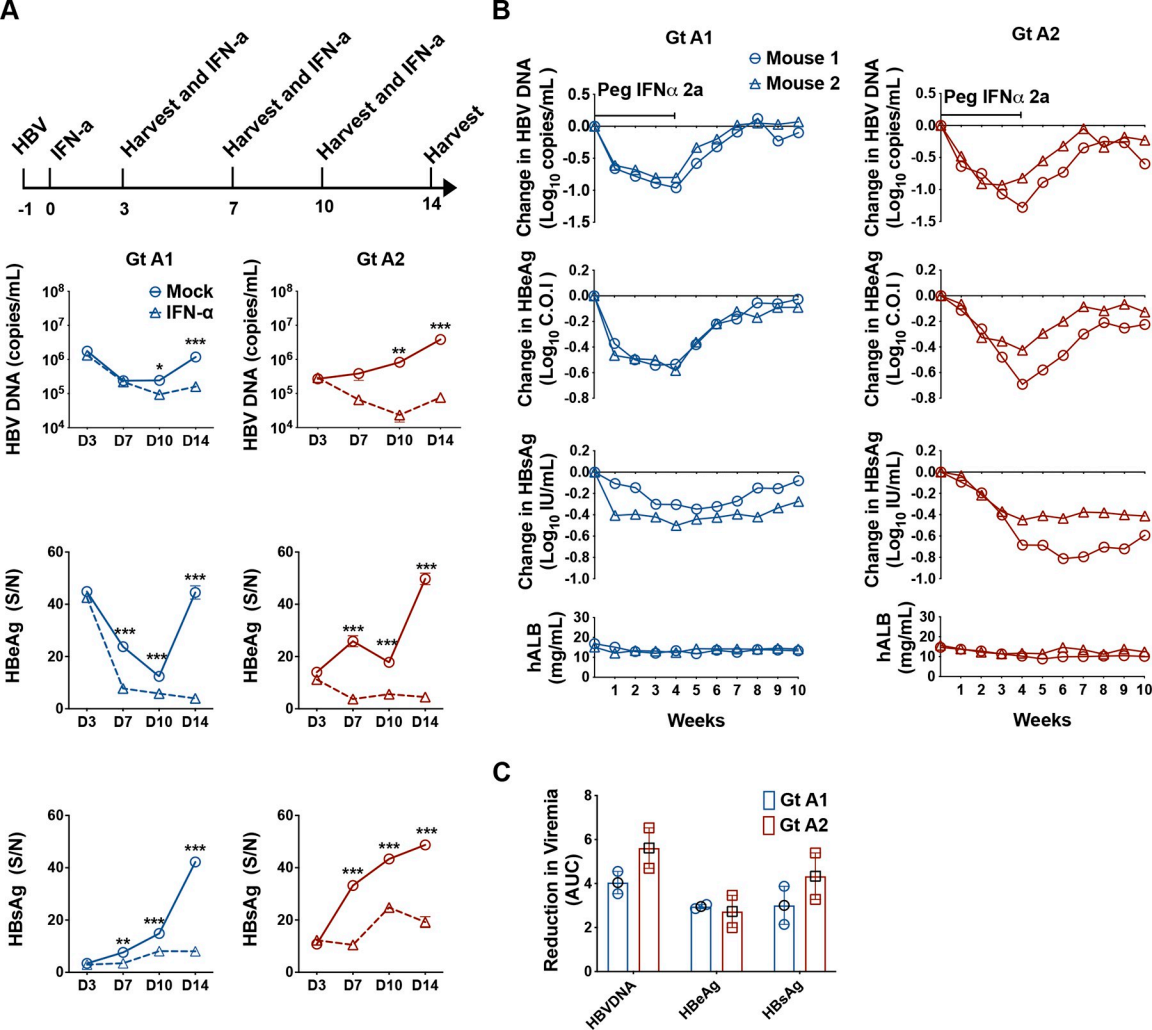
Antiviral activities of hIFN-α2a in HBVcc subtypes A1 and A2 infected PXB-cells and human liver chimeric mice. **(A)** PXB-cells were infected with HBVcc subtypes A1 and A2 at an MOI of 10 (open circles with solid line) and then treated with hIFN-α2a (500 U/mL) for 14 days (open triangles with dotted line). The anti-HBV effect of hIFN-α2a on production of HBV DNA, HBeAg and HBsAg on days 3, 7, 10 and 14 post-treatment were evaluated. Data are shown as means ± SEM of triplicates. *** *P*<0.001; ** *P*<0.01; * *P*<0.05. **(B)** HBVcc-infected human liver chimeric mice (n = 2 per subtype) were treated with pegIFN-α2a (30 μg/kg) at week 13 post-infection and continued for 4 weeks. Mice serum was harvested weekly at indicated time points during the treatment period and continued for 6 weeks after treatment cessation. Changes of HBV DNA, HBeAg and HBsAg levels from the pre-treatment levels (week 13 of infection) are shown. The data of each mouse are denoted by a different symbols & lines. **(C)** The cumulative changes of HBV DNA, HBeAg and HBsAg during 4 weeks of pegIFN-α2a treatment were calculated as area under the curve (AUC) from weeks 1–4 of treatment. Data are shown as means ± SEM of duplicates.

Previous clinical studies suggested that patients infected with different HBV genotypes may have different responses to hIFN-α treatment [2]. We thus evaluated the response of HBVcc A1 and A2 to hIFN-α2a in this model. We treated PXB-cells infected with A1 and A2 (MOI of 10) with 500 IU/mL hIFN-α2a on day 1 post-infection and continued for 2 weeks. As shown in Fig 2A, all HBV markers were suppressed by hIFN-α2a treatment for both the subtypes. Patients with high HBV DNA levels have been reported to respond less well to IFN-α treatment [18]. To assess the antiviral effect of hIFN-α2a in cells with sustained and high level of HBV replication, we tested the effect of hIFN-α2a on day 10 post-infection when HBV DNA level was high and continued the treatment for 7 days. As shown in S2 Fig, hIFN-α2a remained effective in suppressing HBV DNA, HBeAg and HBsAg in both subtype-infected cells.

After establishment of infection in human liver chimeric mice (week 13 post-HBVcc infection), pegIFN-α2a (30 μg/kg) was administered twice weekly for 4 weeks. The mice were then followed for 9 more weeks off treatment. Serum samples were obtained weekly and hAlb, HBV DNA, HBeAg and HBsAg were tested. As shown in Fig 2B, pegIFN-α2a suppressed serum HBV DNA, HBeAg and HBsAg levels in both A1 and A2 infected mice during the treatment period. As expected, all the HBV markers rebounded gradually after the termination of pegIFN-α2a. We determined the area under curve (AUC) of changes in HBV DNA, HBeAg and HBsAg of both subtype-infected mice during the 4-week treatment period (Fig 2C). Overall, suppression of HBV markers was similar between A1 and A2.

### Efficient infection of PXB-cells by HBV A1 and A2 passaged in human liver chimeric mice

We previously showed that HBVcc passaged in the human liver mouse model (HBVmp) infects cell culture more efficiently [17]. Here we examined whether HBV subtypes A1 and A2 also acquire this property after passaging in the human liver chimeric mice. We compared infectivity of HBVmp to HBVcc of A1 and A2 in PXB-cells at the same MOI. As shown in S3 Fig, HBVmp of A1 and A2 infected PXB-cells efficiently with a substantially higher production of HBV DNA, HBeAg, HBsAg and intracellular HBV RNA than the HBVcc of corresponding subtype. Both HBVcc and HBVmp infections of PXB-cells were blocked by Myrcludex B. We sequenced the HBVmp of both subtypes and showed no mutations were acquired during passage in mice.

**Fig 3:**
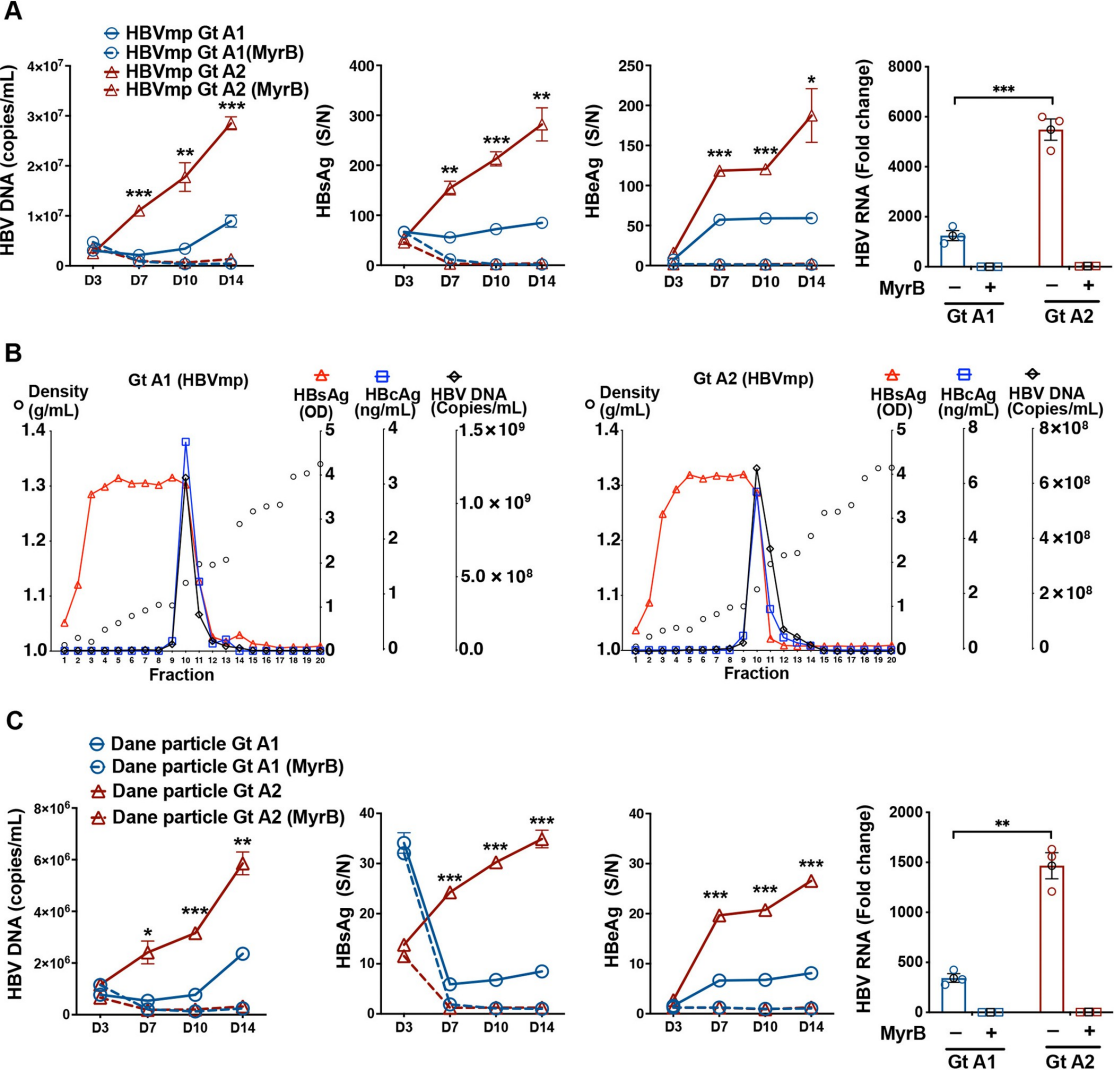
Comparison of HBVmp and Dane particles infection of subtypes A1 and A2. **(A)** PXB-cells were infected with HBVmp of subtypes A1 and A2 at an MOI of 5. Culture media on days 3, 7, 10 and 14 post-infection were harvested and assayed for HBV DNA, HBeAg and HBsAg. PXB-cells were harvested at day 14 post-infection for determination of intracellular HBV RNA. HBV markers were compared between subtypes A1 and A2. **(B)** HBVmp of subtypes A1 (100 μL of 1.2×10^9^ copies/mL) and A2 (100 μL of 1.69×10^9^ copies/mL) stocks were diluted in 400 μL PBS and layered on top of a preformed iodixanol density gradient and subjected to centrifugation density gradient analysis as described in the methods. After centrifugation, 20 fractions (500 μL per fraction) were collected from each gradient. HBsAg, HBcAg and HBV DNA were measured in each fraction accordingly. **(C)** PXB-cells were infected with Dane particles purified from HBVmp (fraction 10) of subtypes A1 and A2 at an MOI of 5. Culture media on days 3, 7, 10 and 14 post-infection were harvested and assayed for HBV markers. PXB-cells were harvested at day 14 post-infection for test of intracellular HBV RNA. HBV markers were compared between subtypes A1 and A2. Statistical analysis was performed with unpaired multiple *t* test. Data are shown as means ± SEM of triplicates. *** *P*<0.001; ** *P*<0.01; * *P*<0.05.

To verify the different replication phenotype of subtypes A1 and A2, we performed a time course analysis of HBVmp infection in PXB-cells and showed consistently higher HBV markers at all time points in A2-infected cells (Fig 3A). We also purified Dane particles (infectious virions) from HBVmp of both subtypes by iodixanol density gradient and infected PXB-cells with the same MOI of fraction 10 (where Dane particles were) of each subtype (Fig 3B). Dane particles of subtype A2 exhibited significantly higher HBV markers than those of subtype A1 (Fig 3C).

### Mechanism of higher replication phenotype of HBV subtype A2

**Fig 4:**
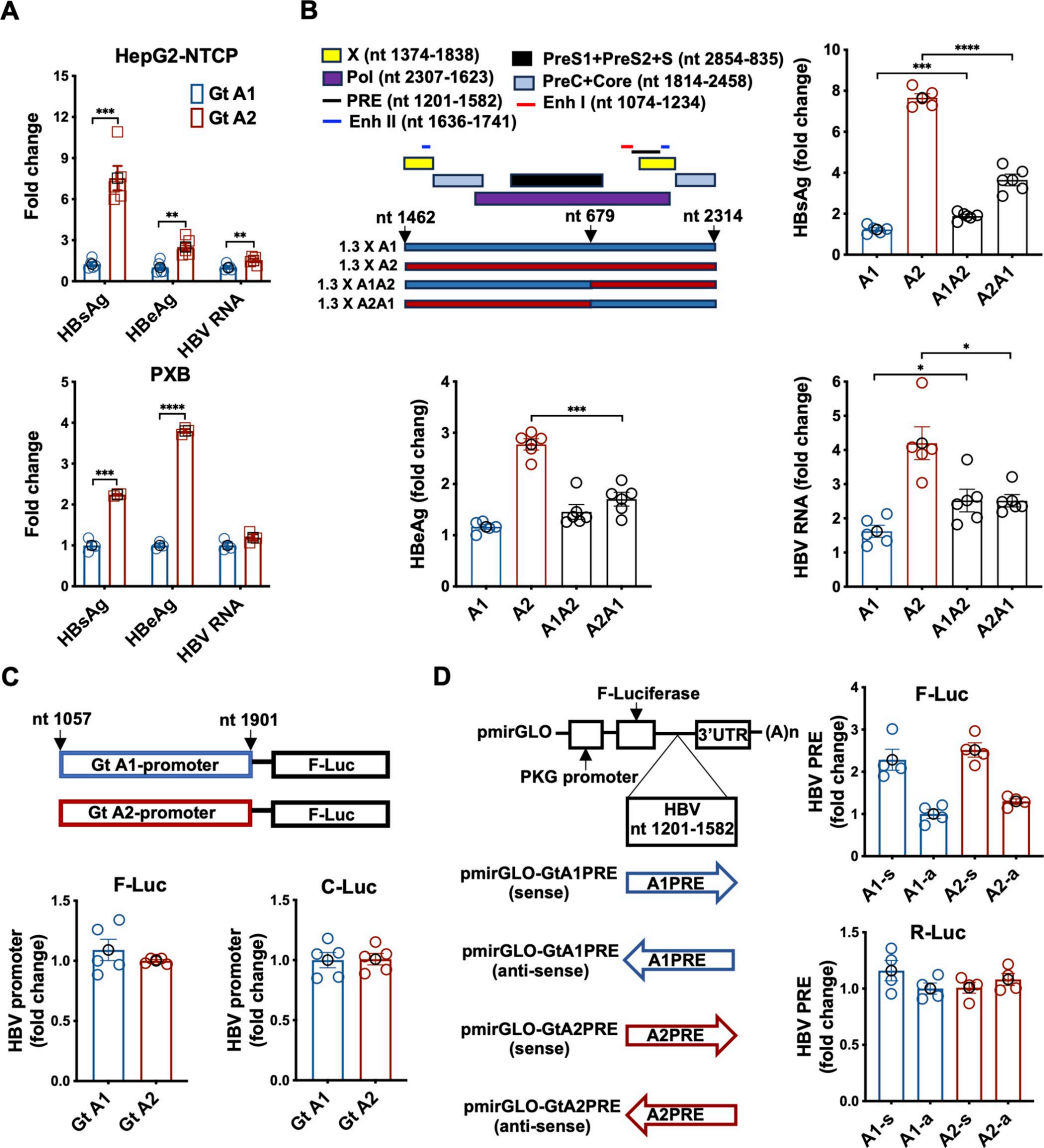
Mechanistic studies of higher replication phenotype of HBV subtype A2. **(A)** 1.3X genome-length HBV subtype A1 or A2 replicative competent construct plasmid was transient transfected into HepG2-NTCP and PXB-cells. Culture media on day 7 after transient transfection were harvested and assayed for HBeAg and HBsAg with ELISA kit. Cells were lysed for HBV RNA determination. Data are shown as mean ± SEM of triplicate or sextuplicate. **(B)** The SpeI-NheI fragments of subtypes A1 and A2 (nt 679–2314) were exchanged to generate the A1A2 or A2A1 chimeras as shown in the schematic diagrams. Different colored boxes represent ORFs and colored lines represent regulatory elements. HBV markers were assayed after 7 days of transfection of chimeras. Data are shown as mean ± SEM of pentaplicate. **(C)** The enhancer I/II/X/core promoter regions of subtype A1/A2 (nt 1057–1901) were amplified and cloned into SacI/HindIII sites of pGL2 luciferase basic. The constructs were co-transfected with pCMV-Cypridina-Luc (transfection control) into HepG2 cells. Three days later after transfection, the transfected HepG2 cells and supernatant were harvested, lysed, and measured for luciferase activities. **(D)** The amplified PREs (nt 1201–1582) of A1/A2 were cloned into the XhoI/XbaI sites of the luciferase reporter vector pmirGLO in both orientations. The arrow shows the orientation of PRE as sense and anti-sense. The pmirGLO construct contains a second gene expression unit, in which R-Luc is driven by SV40 early enhancer/promoter and is used as a control. Two days after plasmid transfection into HepG2 cells, cell lysate and supernatant were harvested and measured for luciferase activities. **** *P*<0.0001; *** *P*<0.001; ** *P*<0.01; * *P*<0.05.

The above HBV markers’ differences between A1 and A2 can be explained by either a higher infectivity or higher replication. To study the underlying mechanism, we transfected replication-competent DNA constructs of A1 and A2 into HepG2-NTCP or PXB-cells and demonstrated a higher production of HBsAg, HBeAg and HBV RNA in A2- than A1-transfected cells (Fig 4A). This finding points to a phenotype of higher viral gene expression by the A2 virus. A1 and A2 show sequence divergence in various locations of the viral genome (S4 Fig) that can affect viral gene expression, such as Enhancer I (Enh I) and Enhancer II (Enh II), X promoter, core promoter, post-transcriptional regulatory element (PRE) or HBx open reading frame (ORF) [19,20].

We next generated chimeric constructs of A1 and A2 (Fig 4B) and demonstrated that the responsible sequences reside between nt 679 to 2314. In the 1.3X HBV construct, the replacement of the 3’ redundant sequences appeared to confer the appropriate intermediate phenotype between A1 and A2. The intermediate phenotype can be explained by the N-terminal redundant sequences that codes a second copy of HBx in the 1.3X HBV construct. Next we reason that the responsible element is either the enhancer/promoter sequences, PRE or HBx. We then generated a construct with either A1 or A2 sequence from nt 1057 to 1901 containing the Enh I, Enh II, X promoter and core promoter driving the luciferase reporter (Fig 4C). The A1 and A2 constructs did not show any appreciable difference, suggesting that the enhancer/promoter region is likely not responsible for the increased gene expression of subtype A2 (Fig 4C).

HBV contains an orientation-dependent RNA element PRE, which has been shown to be required for efficient HBV gene expression [20]. Two highly conserved stem-loops SLα and SLβ are required for PRE function [21]. A1 and A2 show sequence divergence in various locations of the PRE including the crucial motif of SLα of the PRE (S4C Fig). PmirGLO luciferase reporter construct with either A1 or A2 sequence from nt 1201 to 1582 containing PRE was therefore generated to assess the role of PRE (Fig 4D). The sense-orientation constructs of PRE from both A1 and A2 had significantly higher activities than those of anti-sense-orientation, but there was no significant difference between A1 and A2 constructs, indicating that PRE is not responsible for the phenotypic difference (Fig 4D).

### HBx variations are responsible for the higher replication phenotype of HBV subtype A2

**Fig 5:**
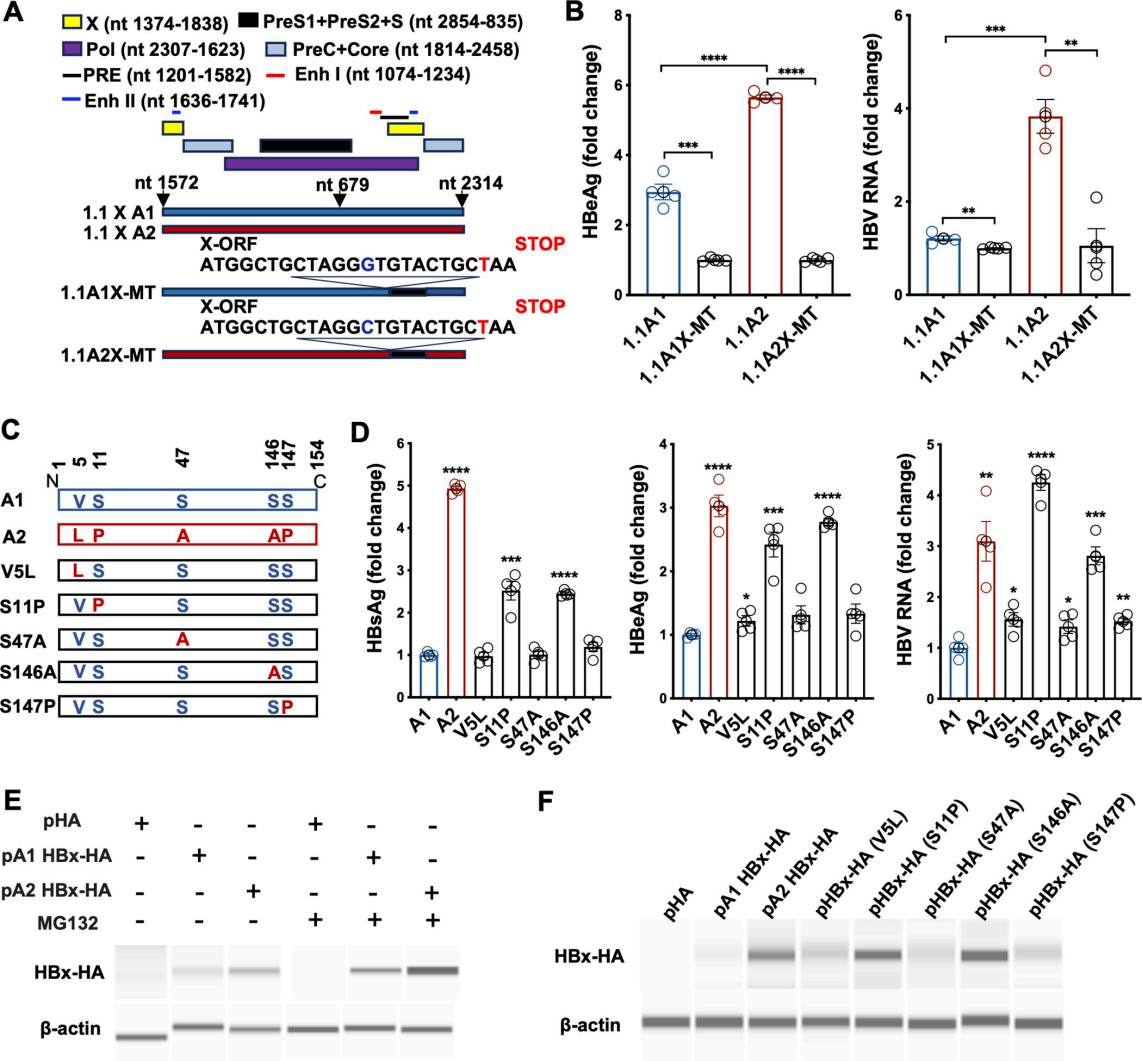
HBx contributes to a higher replication phenotype of HBV subtype A2. **(A)** The 1.1X HBV genotype A1 or A2 replicative competent construct containing only one copy of HBx ORF was generated by deleting the NotI-RsrII fragment of the 1.3X HBV construct. A stop codon mutation was then introduced into the HBx ORF as 1.1A1X-MT and 1.1A2X-MT. **(B)** The 1.1XA1/A2 wildtype and X-mutant plasmids were transfected into HepG2 cells. Two days post-transfection, supernatants were collected for HBeAg assay, and cells were harvested for the quantification of HBV RNA. **(C)** Mutants were introduced into 1.1X A1 construct in the HBx: V5L, S11P, S47A, S146A and S147P. **(D)** HBV markers (HBsAg, HBeAg and HBV RNA) were tested in wildtypes and mutants and compared with wildtype A1. **(E)** HepG2 cells were transfected with plasmid constructs expressing 3xHA-epitope-tag on an empty vector (pHA), HBx subtype A1 (pA1 HBx-HA), and A2 (pA2 HBx-HA). Two days later, cells were treated with or without MG132 proteasome inhibitor (10 μM) for 6 hours , harvested and assayed by WES Western blot. **(F)** HepG2 cells were transfected with plasmid constructs expressing 3xHA-epitope-tag on an empty vector (pHA), HBx subtype A1 (pA1 HBx-HA), A2 (pA2 HBx-HA) and the various mutants. The WES Western blot experiment was repeated three times independently with similar results and unessential lanes were removed from the original blot images. Data are shown as means ± SEM of quadruplicate. **** *P*<0.0001; *** *P*<0.001; ** *P*<0.01; * *P*<0.05.

To further investigate the responsible genetic element, we generated constructs to assess the function of HBx [22,23]. We then inactivated HBx expression coded in the C-terminal sequences by introducing a stop codon TAA in both 1.1X A1 (1.1A1X-MT) and 1.1X A2 (1.1A2X-MT) constructs (Fig 5A). This mutation does not affect any known cis-regulatory elements in the enhancer/promoter region nor affects the polymerase-ORF (synonymous mutation) either [24]. After transfection of these wildtype and mutant plasmids into HepG2 cells, supernatant and cells were collected for HBV markers analysis. The 1.1X A2 showed a higher production of HBeAg and HBV RNA than the 1.1X A1 (Fig 5B), consistent with the data from the 1.3X constructs. Both 1.1A1X-MT and 1.1A2X-MT exhibited much lower levels of HBeAg and HBV RNA, indicating that inactivation of the X-ORF results in lower HBV expression (Fig 5B). Interestingly, 1.1A1X-MT and 1.1A2X-MT did not show any appreciable differences in HBV marker levels, suggesting that the HBx ORF variations likely contribute to the increased HBV replication of subtype A2.

Alignment of A1 and A2 sequences used in this study shows HBx sequence variations at amino acid (aa) 5, 11, 47, 146 and 147 (Figs 5C and S4E). Further comparison of all subtypes A1 and A2 sequences curated from the public database reveals a similar pattern of HBx variations in those locations (S4F Fig). We next generated HBx mutants (V5L, S11P, S47A, S146A and S147P) in subtype A1 and evaluated the effects of these mutations (Fig 5C and 5D). Compared to wildtype A1, the mutants S11P and S146A showed significantly increased HBV markers, almost to the levels of subtype A2. Collectively, these results indicated that these two amino acid variations are responsible for the higher HBV replication phenotype of subtype A2.

To further characterize the functional effects of these HBx variations, we generated HBx expression constructs and transfected them into HepG2 cells. Interestingly, the A2 HBx construct expressed a much higher level of HBx than that of A1 (Fig 5E). It is known that HBx is degraded by the ubiquitin-proteasome degradation pathway [25]. Treatment with proteasome inhibitor MG132 was able to significantly increase the levels of HBx in both A1 and A2 transfected cells (Fig 5E). Testing of individual HBx mutant constructs revealed that the S11P and S146A mutants expressed a higher level of HBx (Fig 5F), supporting that these two variations are indeed responsible for the phenotype.

**Fig 6:**
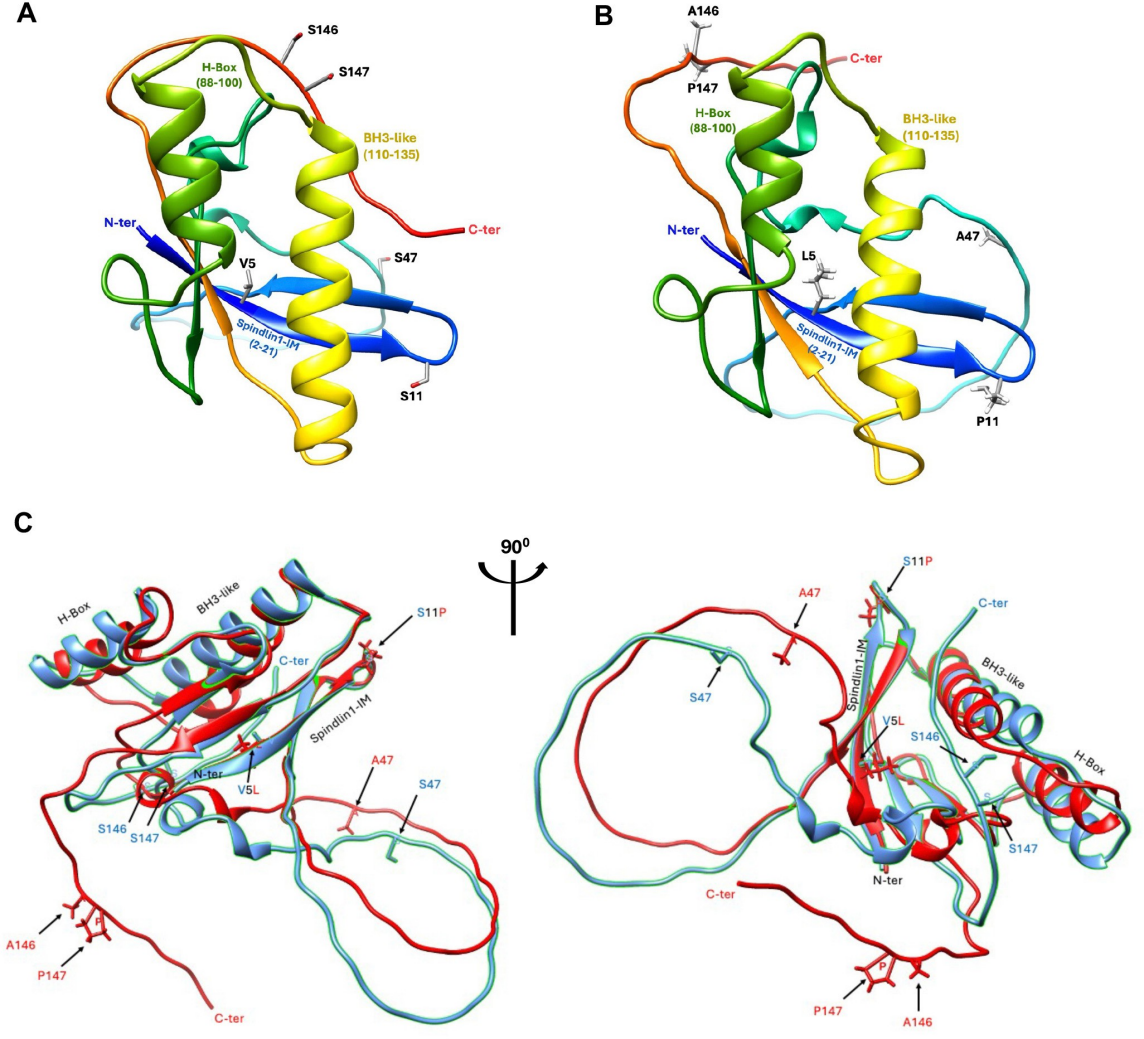
**Alphafold2 models of HBx subtypes A1 (A) and A2 (B)**. **(A, B)** the previous defined HBx domains (Spindlin1-Interactive Motif, aa 2–21; H-Box, aa 88–100; BH3-like domain, aa 110–135) and the putative N and C-termini of the protein are shown. The five aa variations of A1 and A2 HBx proteins are shown as side chains sticks (grey, carbon; red, oxygen; white, hydrogen). **(C)** Superposition of AlphaFold2 models of HBx subtype A1 (blue) and subtype A2 (red). The five aa variations of the subtypes A1 and A2 in their respective colors are depicted.

Finally, we generated AlphaFold2 (AF2) models of the two subtypes of HBx protein (Fig 6A and 6B). Previously defined structural motifs within HBx including a Spindlin1-interacting domain (aa 2–21), an H-box motif (aa 88–100, interacting with DDB1), and a BH3-like motif (aa 110–135, interacting with Bcl-2) are shown. Superposition of the A1 and A2 models reveal that these structural motifs are similarly aligned (Fig 6C). Interestingly, the C-terminal peptides diverge between the A1 and A2 models, probably because of the aa 146 and 147 variations (S146A, S147P).

## Discussion

HBV genotypes have distinct geographical distributions, routes of transmission, virological features and clinical characteristics. Although these differences could be explained by racial/ethnic background, environment or other confounding factors, HBV genotypes and sequence variations may play an important but yet-to-be defined role in the pathogenesis of liver disease. We have recently established these genotypes cell clones and explored their infection courses, virological behaviors and interferon responses in cell culture and animal models [17]. HBV subtypes A1 and A2, despite being classified within one genotype, appear to have distinct virologic and clinical features [12,13,15]. Here we compared the two subtypes, A1 and A2 in cell culture and animal models. We established cell lines producing high-titer HBV subtypes A1 and A2 and showed their infectivity in PXB-cells. In addition, these viruses can be successfully passaged in human liver chimeric mouse models (human hepatocyte-engrafted *Alb-uPA/Scid* mice). The passaged viruses (HBVmp) are highly infectious in PXB-cells. We also compared the infectivity, replication and interferon treatment response of A1 and A2 using these models and demonstrated the mechanism responsible for the different replication phenotypes.

HBV DNA levels in subtype A1 carriers have been reported to be significantly lower than those of subtype A2 carriers, in both HBeAg-positive and negative phases [15]. Subtype A1 has more divergent sequences comparing to A2, which may affect both the replication of the virus and expression of viral proteins [10,11,16]. Here we showed that subtype A2 indeed has higher HBsAg, HBeAg and HBV DNA levels than that of subtype A1 after infection of PXB-cells with HBVcc or HBVmp at the same MOI. Mechanistic investigations of the sequence divergence between the two subtypes indicated that A2 indeed exhibits higher gene expression levels. Consistent with what we found *in vitro*, A1 and A2 also exhibited different characteristics of infection in the mouse model. Our data in these cell and animal models showing higher HBV replication in subtype A2 than A1 are consistent with the observations from clinical reports [15], further supporting the use of this model system to explore clinically relevant questions on HBV genotypes.

Patients with subtype A1 infection exhibited a relatively higher rate of seroconversion from an HBeAg-positive to an HBeAg-negative state [12]. The frequency of HBeAg positivity in subtype A1 carriers is significantly lower than carriers infected with subtype A2, especially in carriers younger than 30 years [15]. Subtype A1 has been associated with a high rate of HCC in sub-Saharan Africa [13]. In South Africa, HCC occurs in younger men who are anti-HBe positive and often do not have accompanying cirrhosis. In contrast to A1, liver cancer associated with A2 is found primarily in older individuals. So far there has been no clear explanation for the different clinical characteristics between subtypes A1 and A2. Some studies have speculated that molecular traits or virological features affecting HBV gene expression and/or replication may contribute to distinct clinical presentations of subtype A1 vs A2-infected patients [10,11]. Higher viral replication may promote chromosomal integration of abortive replicative intermediates and hence contribute to hepatocarcinogenesis [26,27]. However, host and environmental factors likely play a more prominent role in HCC development among the populations infected with A1 (Africa) vs A2 (Europe). Since our *in vivo* and *in vitro* models mimic the process of HBV gene expression and replication in patients, we believe such systems are valuable in addressing this question of HBV-induced HCC.

Clinical studies indicate that HBV patients with genotype A2 infection respond better to interferon than other genotype-infected patients [28]. Scant data are available on the response of IFN to subtype A1. Previous studies have used transient transfection system to study subtype A1 and A2 in cell culture [29], but the data may not reflect biological infection. Using infected PXB-cells and chimeric human mice, we found that A1 and A2 responded equally well to PegIFN-α2a. However, caution must be exercised when extrapolating the results of *in vitro* and *in vivo* experimental models to humans, because duration of infection, host genetics, immune response and environmental factors likely play a dominant role in the disease progression and treatment response in humans.

The distinct replication phenotypes associated with subtypes A1 and A2 are likely a result of their sequence variations. As mentioned above, the nucleotide sequence of subtype A1 differs from A2 by about 4%. Our systematic analyses of all HBV genetic elements suggested that the *HBx* gene is responsible for the increased HBV replication phenotype associated with A2. The HBx protein has been implicated in diverse functions and shown to interact with various host proteins [30]. Alignment and comparison of amino acid sequences of all available A1 and A2 strains indicate several locations with major sequence variations at aa 11, 31, 47, 147 and 148 (S4F Fig). These variations do not fall within the previously reported sequence of HBx (aa 88–100) that interacts with its well-known interacting cellular protein DDB1 [31,32]. Recently other regions of HBx, such as aa 2–21, aa 103–140 and 129–155 were shown to interact with other cellular proteins associated with HBx function [33–35]. Our study showed HBx aa 11 and 146 variations are responsible for the different replication phenotypes of subtypes A1 and A2 based on distinct expression levels of HBx protein. We speculate that the two mutations might stabilize the HBx protein by reducing its degradation by the ubiquitin-proteasome pathway. HBx hijacks Smc5/6 complex and directs it to the Cullin 4 Ring ligase machinery for ubiquitination and degradation by the proteasome [36]. It would be interesting to compare these HBx mutants in the context of Smc5/6 degradation. Future studies will be necessary to address this question. Since proteasome inhibitor MG132 couldn’t totally relieve the different expression of HBx, other mechanisms might play a role.

HBx has 154 amino acid residues that have been proposed to contain an N-terminal negative regulatory region (aa 1 to 50) and a C-terminal transactivation region (aa 51 to 154) [33]. HBx is known to transcriptionally regulate HBV cccDNA via interaction with various epigenetic and transcriptional factors [37]. Intriguingly, the two amino acid residues (aa 11 and 146) responsible for the replication phenotype between A1 and A2 locate at distinct regions. Using AF2, we model HBx proteins of the A1 and A2 subtypes, which show the three previously defined structural domains (Fig 6).

The Spindlin1 interactive motif (Spindlin1-IM), consisting of a highly conserved N-terminal (2–21) of HBx, forms 2 parallel beta-sheets (β1 and β2) (PDB 8GTX) and interacts with Tudor 3 domain of Spindlin1 [33]. Second, the H-Box motif of HBx (aa 88–100) forming a conserved alpha-helix (PDB 3I7H) interacts with the beta propeller C of DDB1 [38]. Finally, the BH3-like motif (aa 110–135) of HBx forms an alpha-helix [39] and binds to Bcl-2 and Bcl-xL [40,41]. The superposition of the two subtypes A1 and A2 showed that most of the models superpose well (Fig 6C). The serine-proline variations at aa 11 position, which is serine in A1 and proline in A2 (S4E and S4F Fig), falls within the Spindlin1-interaction domain (aa 2–21), such a variation may account for the different activities of the corresponding HBx proteins, as serine can form hydrogen bond with other amino acids and proline is known to disrupt the structure of a peptide. Based on the published structure of HBx (aa 2–21) in complex with Spindlin1 (Tudor 3 domain, aa 50–262) [33], aa 11 appears to be in a flexible region between the two β-sheets of this HBx domain. Thus, it is not clear whether this amino acid is involved in interaction with the Spindlin1. The serine to proline mutation, however, could alter either intramolecular or intermolecular interactions that may affect the stability of HBx. The structural models of A1 and A2 starts to diverge around the S47A position. Interestingly, the C-terminal ends of the two models spread in opposite directions. We speculate that this is probably due to the aa 146–147 variations, which are serine-serine in A1 and alanine-proline in A2, respectively (S4E and S4F Fig). At present, it is not clear whether this structural divergence may account for the different degradation rate by the ubiquitin-proteasome pathway. Further studies are necessary to define the structural effects of these variations and the associated mechanism.

The lack of a known structure and the tendency to aggregate within cells, together with a low expression level upon HBV infection, hamper the study of fundamental biological properties of HBx. Also, HBx studies in the context of natural HBV infection represent a major challenge because of limited and inefficient cell culture-based infection systems and the absence of specific and sensitive HBx antibodies [42]. Our study here opened a door for further HBx exploration in these models. While studying single clones may not be representative of the overall genetic diversity of HBV, the observed phenotypes can then be extrapolated to the larger sequence database to determine whether the identified variations are indeed relevant to the sequence variations among diverse HBV genotypes. Here we compare all subtypes A1 and A2 sequences curated from the public database and show a similar pattern of HBx variations that are representative by our clones.

Our previous study of all HBV genotypes demonstrated divergent replication phenotypes in both cell culture and human liver chimeric model [17]. It is particularly interesting that genotype B appears to replicate the highest and genotype C the lowest. Sequence alignment of all genotypes reveal many amino acid variations within the *HBx* gene, raising the intriguing possibility that HBx polymorphisms may account for part of the diverse virological behaviors and disease manifestations of HBV infections. Future studies of HBx sequence polymorphisms among various HBV genotypes/strains may provide important insight into the diverse functions of HBx.

In conclusion, the generation of these replication competent clones provides an important tool in the functional characterization of HBV genotypes. Unique features of HBV, including its narrow virus-host range, its hepatocyte tropism, and diverse genotypes are major challenges in the development of suitable *in vivo* and *in vitro* model systems to recapitulate the complete biology of HBV infection and to test antiviral strategies. Our study provides a valuable platform for these purposes. The surprising and intriguing finding of subtype A2 having a higher replication phenotype based on polymorphisms in the *HBx* gene provides the first indication that sequence variations, specifically in *HBx* gene here and possibly elsewhere on the genome, may underpin the diverse clinical phenotypes of HBV infections. Our study underscores the usefulness of this platform in investigating HBV biology. Further studies are necessary to comprehensively explore the role of viral sequence variations in HBV infection and disease manifestation.

## Methods

### Ethics statement

Experiments with mice were approved by the Ethics Review Committee for Animal Experimentation of the Graduate School of Biomedical Sciences, Hiroshima University (A18-64).

### Cell culture

HepG2 cells were maintained in Dulbecco’s Modified Eagle Medium (DMEM) with L-glutamine & sodium pyruvate (Corning, VA, USA), 100 U/mL penicillin (Corning, VA, USA) and supplemented with 10% fetal bovine serum (Sigma, MO, USA). HepG2-NTCP cells (provided by Prof. Ulrike Protzer) were cultured in DMEM (Corning, VA, USA) with 10% FBS, 100 U/mL penicillin (Thermo Fisher Scientific, MA, USA) and 30 μg/mL blasticidin (Thermo Fisher Scientific, MA, USA). Human hepatocytes passaged and expanded in *Alb-uPA/Scid* mice [43], provided by Dr. Takeshi Saito (Keck School of Medicine of USC, University of Southern California) or PhoenixBio, NY, USA (PXB-cells) were cultured in DMEM with 1 g/L glucose, L-glutamine & sodium pyruvate (Corning, VA, USA), 100 U/mL penicillin (Thermo Fisher Scientific, MA, USA), 15 ug/mL L-Proline (MP Biomedicals, USA), 0.25 ug/mL insulin (Sigma, MO, USA), 50 nM Dexamethasone (Sigma, Aldrich, USA), 5 ng/mL EGF (Sigma, Aldrich, USA), 0.1 mM L-ascorbic Acid 2-Phosphate (Wako Pure Chemical Industries, Ltd, USA), 2% DMSO and supplemented with 10% fetal bovine serum (Sigma, MO, USA). Both cells were cultured on collagen I (Gibco, NY, USA) coated plates or flasks.

### Construction of stable HBV-producing cell lines

1.3X genome-length of HBV subtype A1 (NCBI accession number KU736919) or A2 (NCBI accession number AF305422) was inserted into the plasmid pcDNA3.1/Hygro (**+**) with the CMV promoter removed, as described in S1 Fig. The full-length DNA of A2 has been described previously [17]. The pcDNA3.1/Hygro (+) /CMV (-) /1.3X HBV was linearized by LguI and purified for transient transfection into HepG2 cells in 10 cm dish using the Lipofectamine 2000 transfection reagent (Thermo Fisher Scientific, MD, USA). 24 hours later, the cells were replated in 10 cm dishes at a 1:30 dilution. Two days after splitting, the cells were cultured in DMEM containing 500 μg/mL hygromycin B (Thermo Fisher Scientific, CA, USA) and the medium was changed every other day for about five weeks until no visible detached cells and clones formed well. The spot clones were collected using Clone Cylinder (EMD Millipore, MA, USA) and transferred to 96-well plates. Once the cells were grown up to around 90% confluent, the supernatants were collected for the detection. Clones secreting the highest levels of HBV DNA and/or HBeAg among various genotypes were chosen for further study.

### HBV production and infection assay

High-titer HBV was prepared from cell clones stably producing and secreting the virus in the medium as described previously [44]. Cell clones expressing A1 and A2 were grown in DMEM (Corning, VA, USA), 100 U/mL penicillin, and supplemented with 10% fetal bovine serum (Sigma, MO, USA). After expansion, the cells were transferred and grown in HYPERFlask Vessels (Corning, NY, USA) with culture medium mentioned above respectively and supplemented with 1.8% DMSO (Sigma Aldrich, MO, USA). The supernatants were purified using heparin column (Cytiva 5mL HiTrap Heparin HP, Cytiva Life Sciences, MA, USA) and concentrated using Centricon Plus-70 (EMD Millipore, MA, USA) and tested HBV DNA by qPCR. For infection, inoculation of PXB-cells was performed with indicated multiplicity of infection (MOI) in indicated cell culture medium containing 5% PEG8000 (Sigma Aldrich, MO, USA) for 24 hours. After incubation, cells were washed with PBS 1X (Corning, VA, USA) three times and cultured in indicated medium. Extracellular HBV DNA, HBsAg and HBeAg secretions in the medium were evaluated at days 3, 7, 10, and 14 after infection. To determine antiviral activity, IFN-α (500 IU/mL) was added at indicated time points. If indicated, Myrcludex B (500 ng/mL) was added as an entry inhibitor.

### HBV infection of human liver *chimeric Alb-uPA/Scid* mice

HBV infection *in vivo* was conducted using chimeric mice with humanized livers by transplantation of human hepatocytes into the spleen of albumin promoter-driven urokinase-type plasminogen activator in severe combined immunodeficient mice (*Alb-uPA/Scid*) as previously described [45]. HBVcc (1×10^6^ copies) was injected into mice through tail vein. PegIFN-α2a (30 μg/kg) was administered at week 13 post-HBVcc infection, and the treatment continued for 4 weeks, after which we continued to monitor the mice for 9 additional weeks. Mice serum was obtained weekly for measurements of human albumin (hAlb) and various HBV markers.

### Generation and transfection study of chimeric A1/A2 constructs

The chimeric A1/A2 constructs were generated as described previously [17]. The chimeric A1A2/A2A1 constructs were generated by changing an SpeI/NheI HBV fragment from nt 679 to 2314 between Gt A1 and A2. DNA transfection by the Lipofectamine 3000 (Thermo Fisher Scientific, MA, USA) was performed. For analysis, the supernatant and cells were harvested 7 days after transfection. To verify the results, all transfection experiments were routinely performed in triplicate at a minimum.

To determine the transcriptional activity of the HBV subtype A1 or A2 promoter, the enhancer I/II/X/core promoter regions of subtype A1/A2 (nt 1057–1901) were amplified using primers (5’-ATGAGCTCCCTTTGTATGCATGTAT-3’/ 5’-GCAAGCTTGCCCCAAAGCCATCCAA-3’) for subtype A1 and (5’-ATGAGCTCCCTTTGTATGCATGTAT-3’ / 5’- GCAAGCTTGCCCCAAAGCCACCCAA-3’) for subtype A2 and cloned into SacI/HindIII sites of pGL2 F-luciferase basic (without any promoter) or promoter reporter vector. pCMV-Cypridina-Luc was used as transfection efficiency control. The post-transcriptional regulatory element (PRE) of A1 or A2 (nt 1201–1582) were amplified using 5’ primer 5’-CCGCTCGAGCGGACTGGCTGGGGCTTGGC-3’ (sense PRE construct) or 5’-GCTCTAGAGCACTGGCTGGGGCT-3’ (anti-sense PRE construct), and 3’ primer 5’-GCTCTAGAGCGCACACGGACCGGCAGAT-3’ (sense PRE construct) or 5’-CCGCTCGAGCGGGCACACGGACCGGCAGAT-3’ (anti-sense PRE construct). The XhoI/XbaI sites in the primers are underlined accordingly. The amplified PREs of A1/A2 were cloned into the XhoI/XbaI sites of the luciferase reporter vector pmirGLO in both orientations (Promega, Madison, USA) [46]. The plasmids were transfected into HepG2 cells using Lipofectamine 3000 (Thermo Fisher Scientific, MA, USA). Two or three days after transfection, the HepG2 cells and supernatant were harvested, lysed, and measured exactly as instructed for F-Luc (AAT Bioquest, CA, USA) and C-Luc (Thermo Fisher Scientific, MA, USA) assays.

To further investigate the responsible genetic element, we generated constructs to assess the function of HBx. 1.1X genome-length HBV subtype A1 or A2 replicative competent construct containing only one copy of HBx open reading frame (ORF) was generated by deleting the NotI-RsrII fragment (the 5’ redundant sequence) of the 1.3X genome-length HBV construct. HBx mutants (stop codon at amino acid position 8) of the 1.1X HBV were amplified using primers 5’-AGGGTGTACTGCTAACTGGATTCTT-3’ and 5’-AAGAATCCAGTTAGCAGTACACCCT -3’ for A1X-MT, and 5’-AGGCTGTACTGCTAACTGGATCCTT-3’ and 5’-AAGGATCCAGTTAGCAGTACAGCCT-3’ for A2X-MT with QuikChange II XL Site-Directed Mutagenesis Kit (Agilent Technologies, CA, USA). The primers used for other HBx mutants are shown in S1 Table. The plasmids were transfected into HepG2 cells using Lipofectamine 3000 (Thermo Fisher Scientific, MA, USA). Two days later after transfection, HepG2 cells and supernatant were harvested for HBV markers test.

### Generation of HBx expression constructs and evaluation by WES Western blot

The DNA fragment encoding C-terminal HA tagged HBx was amplified from the cDNA of wildtypes A1 and A2, and mutant A1 using primers 5’-CTAGCTAGCTAGATGGCTGCTAGG-3’ and 5’-CCGCTCGAGCGGTCCGGCAGAGGTGAA-3’ with NheI and XhoI sites underlined. The amplified HBX fragments were cloned into NheI/XhoI sites of vector pCMV6-AC-3HA with C-terminal 3XHA tag (OriGene, MD, USA). HepG2 cells were transfected with an empty vector (pHA) or various HBx constructs (pHBx-HA). Two days later, cells were treated with or without MG132 proteasome inhibitor (Sigma, MO, USA) at 10 μM for 6 hours. Cells were then lysed with protein lysis RIPA buffer (Thermo Fisher Scientific, MA, USA) containing protease inhibitor cocktail (Sigma, MO, USA) according to manufacturer’s protocol. Proteins were then quantified with Pierce BCA assay kit (Thermo Fisher Scientific, MA, USA). All samples were loaded with the same amounts of proteins at 750 μg/ml. Western blots were performed with Protein Simple WES Western Blot System (Bio-Techne) and followed manufacturer’s protocol. The ProteinSimple 12–230 kDa separation module (Thermo Fisher Scientific, MA, USA) was used. The primary antibodies anti-HA (Thermo Fisher Scientific, MA, USA) and anti-β-actin (Abcam, Cambridge, UK) were used to detect HBx-3xHA and β-actin (used as loading control) respectively. The secondary antibodies anti-mouse and anti-rabbit detection modules (Thermo Fisher Scientific, MA, USA) were used. Analyses of western blot were performed on the Compass for Simple Western software.

### ELISA

HBeAg and HBsAg in cell culture supernatants was measured by ELISA kits (Creative-Diagnostics, NY, USA) according to the protocol. Samples outside the assay linear range were diluted and re-assayed. Quantification of mice serum HBsAg and HBeAg was performed using Abbott Architect platforms (Abbott, Diagnostic Division, Ireland) as recommended by the manufacturer. The plates were read in a MiniMax 300 Imaging Cytometer system using SoftMax Pro7.

### Real time quantitative PCR

Real time qPCR was performed using SYBR green chemistry with LightCycler 480 System II (Roche Diagnostics Corporation, IN, USA). The primers sequences for HBV DNA test are 5’-GCTGGATGTGTCTGCGGC-3’ and 5’-GAGGACAAACGGGCAACATAC-3’.

### Iodixanol density gradient analysis

As described previously [17], iodixanol layers were prepared as 10%, 20%, 50%, 80% and 100% from top to bottom. Viral samples were layered on top of the iodixanol gradient and centrifuged at 30,000 rpm for 18h at 4°C in a SW41 rotor with brake of 0. Fractions were collected carefully from the top of gradient in sequence without any mixing between different layers. The density, HBsAg, HBcAg and HBV DNA were measured accordingly for each fraction.

### AlphaFold2 modeling

We generated 5 predicted structures of HBx of subtypes A1 and A2 using AlphaFold2 [47] based on the sequences of HBx of subtypes A1 and A2 in this study. This work utilized the computational resources of the NIH HPC Biowulf cluster (http://hpc.nih.gov). We superposed various models of subtypes A1 and A2 by using UCSF Chimera [48].

### Statistical analysis

Statistical analysis was performed using Graphpad Prism (version 10.3.0). The standard error of mean (SEM) was calculated from the average of at least 3 independent replicates. Data are shown as mean ± SEM, using Student’s unpaired two-tailed *t* test to evaluate statistical difference. *P* values < 0.05 were considered significant difference.

## Supporting information

S1 Fig Establishment of cell lines producing HBVcc of subtype A1 or A2. 1.3X genome length of HBV subtype A1 or A2 was inserted into the plasmid pcDNA3.1/Hygro (**+**) with the CMV promoter removed. After transfection of HBV genomes of subtype A1 or A2 into HepG2 cells and selection with hygromycin B, clones with highest HBV titers were chosen as candidates of stable cell lines for study. The experimental scheme was generated from BioRender. *We noted two-nucleotide differences, nt 1762 (A to T) and nt 1764 (G to A), comparing to the original sequence in NCBI, when we sequence-confirmed the clone. The detailed information of subtype sequences could be found through Reference [49], Reference [50] and their accession numbers.
(TIF)

S2 Fig Anti-HBV activity of late-stage treatment with hIFN-α2a on HBV subtypes A1 and A2 infected PXB-cells. PXB-cells were infected with HBV subtypes A1 and A2 in triplicates at an MOI of 10 (open circles with solid line). On day 10 post-viral infection, cells were treated with hIFN-α2a (500 IU/mL) for 7 days (open triangles with dotted line). The anti-HBV effects of hIFN-α2a on secretion of HBV DNA, HBeAg and HBsAg on days 10, 14 and 17 post-infections were evaluated and compared to untreated samples (Mock). Statistical analysis was performed with the unpaired multiple *t* test. Data are shown as mean ± SEM of triplicates. *** *P*<0.001; ** *P*<0.01; * *P*<0.05.
(TIF)

S3 Fig Comparison of HBVmp and HBVcc infection of subtypes A1 and A2. PXB-cells were infected with HBVcc and HBVmp (harvested from each infected mouse) at an MOI of 10. Myrcludex B (MyrB) treatment was used as negative control. Culture media on day 14 post-infection were harvested and assayed for HBV DNA, HBeAg and HBsAg. PXB-cells were harvested for determination of intracellular HBV RNA. Error bars indicate mean ± SEM of triplicates.
(TIF)

S4 Fig Sequence divergence in various regions of the subtypes A1 and A2 HBV genome. **A-E,** The sequence of Enhancer I/X promoter, Enhancer II/Core promoter, PRE, HBX ORF and HBx protein were compared between subtypes A1 and A2. The different DNA bases/amino acids were highlighted with red. **F,** Weblogo sequence alignment of HBx between all A1 and A2 sequences. The sequences of the HBx of all available HBV subtypes A1 and A2 were obtained from the NCBI database. Generated alignments and sequence conservation were visualized and calculated with the Mafft Web Service in Jalview 2.11.2.7 and Berkeley Weblogo 3 programs. The value n represents the number of HBV sequences of each genotype included in the alignment. In the alignment figure, hydrophilic amino acids were marked red, neutral amino acids black, and hydrophobic amino acids blue. Major different amino acids were indicated with red arrows above the alignment.
(TIF)

S1 Table Primers for HBx mutation. (DOCX)
