# Supplementary figures and images for: Hepatitis B virus genotypes A1 and A2 have distinct replication phenotypes due to polymorphisms in the HBx gene
Source: PLoS Pathog. 2025 Jan 9;21(1):e1012803. doi: 10.1371/journal.ppat.1012803 (PMC11717313; doi:10.1371/journal.ppat.1012803)

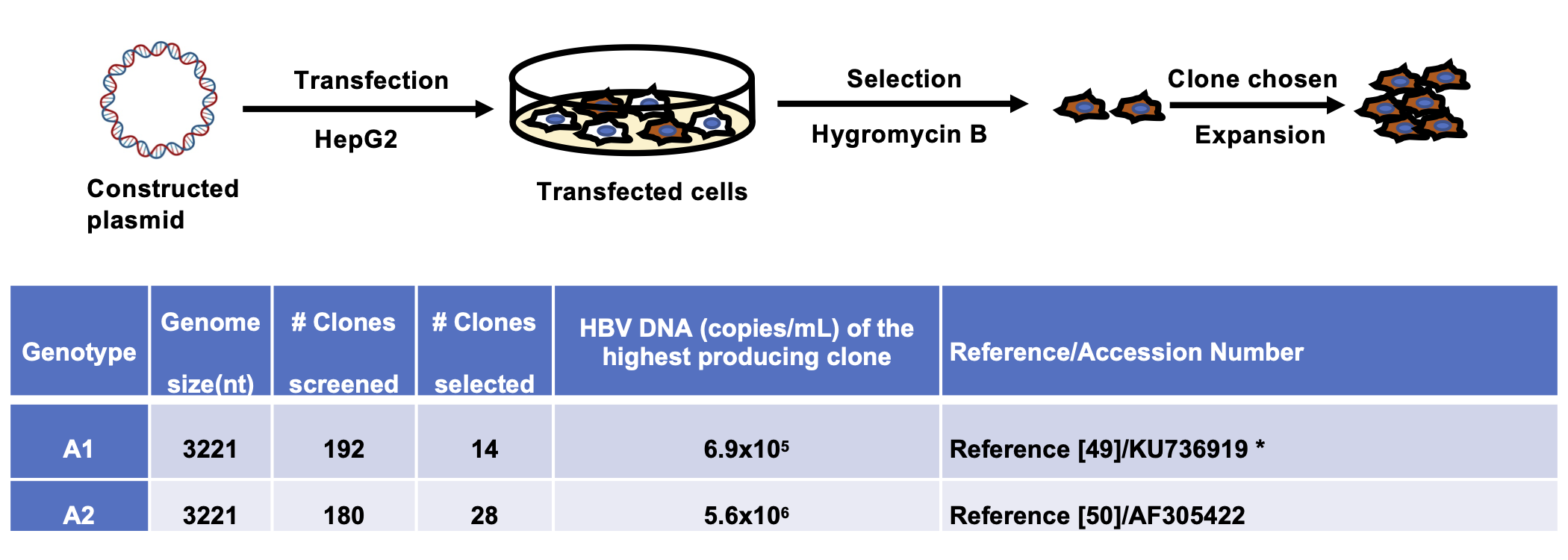

Supplement: S1 Fig — 1.3X genome length of HBV subtype A1 or A2 was inserted into the plasmid pcDNA3.1/Hygro (+) with the CMV promoter removed. After transfection of HBV genomes of subtype A1 or A2 into HepG2 cells and selection with hygromycin B, clones with highest HBV titers were chosen as candidates of stable cell lines for study. The experimental scheme was generated from BioRender. *We noted two-nucleotide differences, nt 1762 (A to T) and nt 1764 (G to A), comparing to the original sequence in NCBI, when we sequence-confirmed the clone. The detailed information of subtype sequences could be found through Reference [49], Reference [50] and their accession numbers. (TIF) [file ppat.1012803.s001.tif]

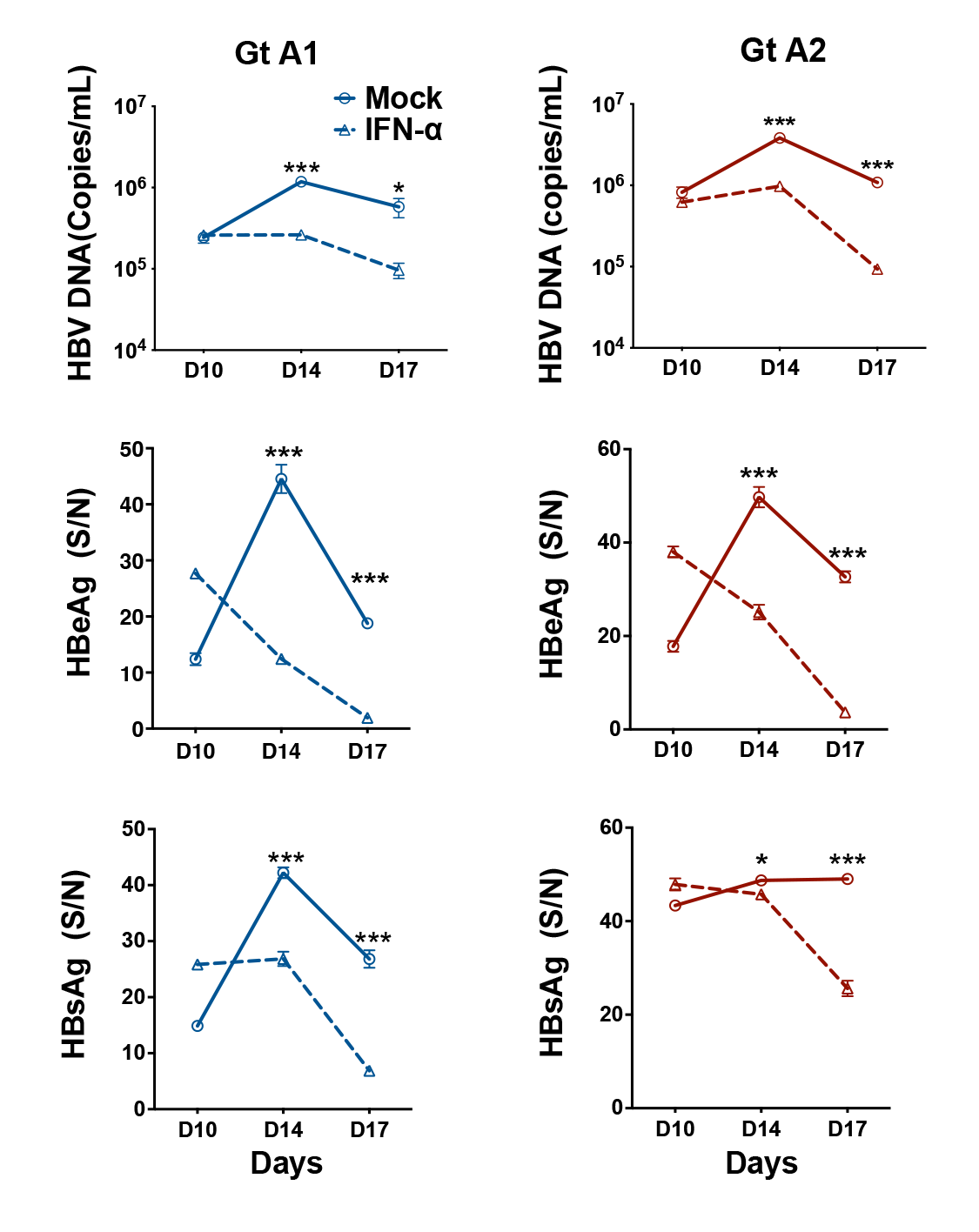

Supplement: S2 Fig — PXB-cells were infected with HBV subtypes A1 and A2 in triplicates at an MOI of 10 (open circles with solid line). On day 10 post-viral infection, cells were treated with hIFN-α2a (500 IU/mL) for 7 days (open triangles with dotted line). The anti-HBV effects of hIFN-α2a on secretion of HBV DNA, HBeAg and HBsAg on days 10, 14 and 17 post-infections were evaluated and compared to untreated samples (Mock). Statistical analysis was performed with the unpaired multiple t test. Data are shown as mean ± SEM of triplicates. *** P<0.001; ** P<0.01; * P<0.05. (TIF) [file ppat.1012803.s002.tif]

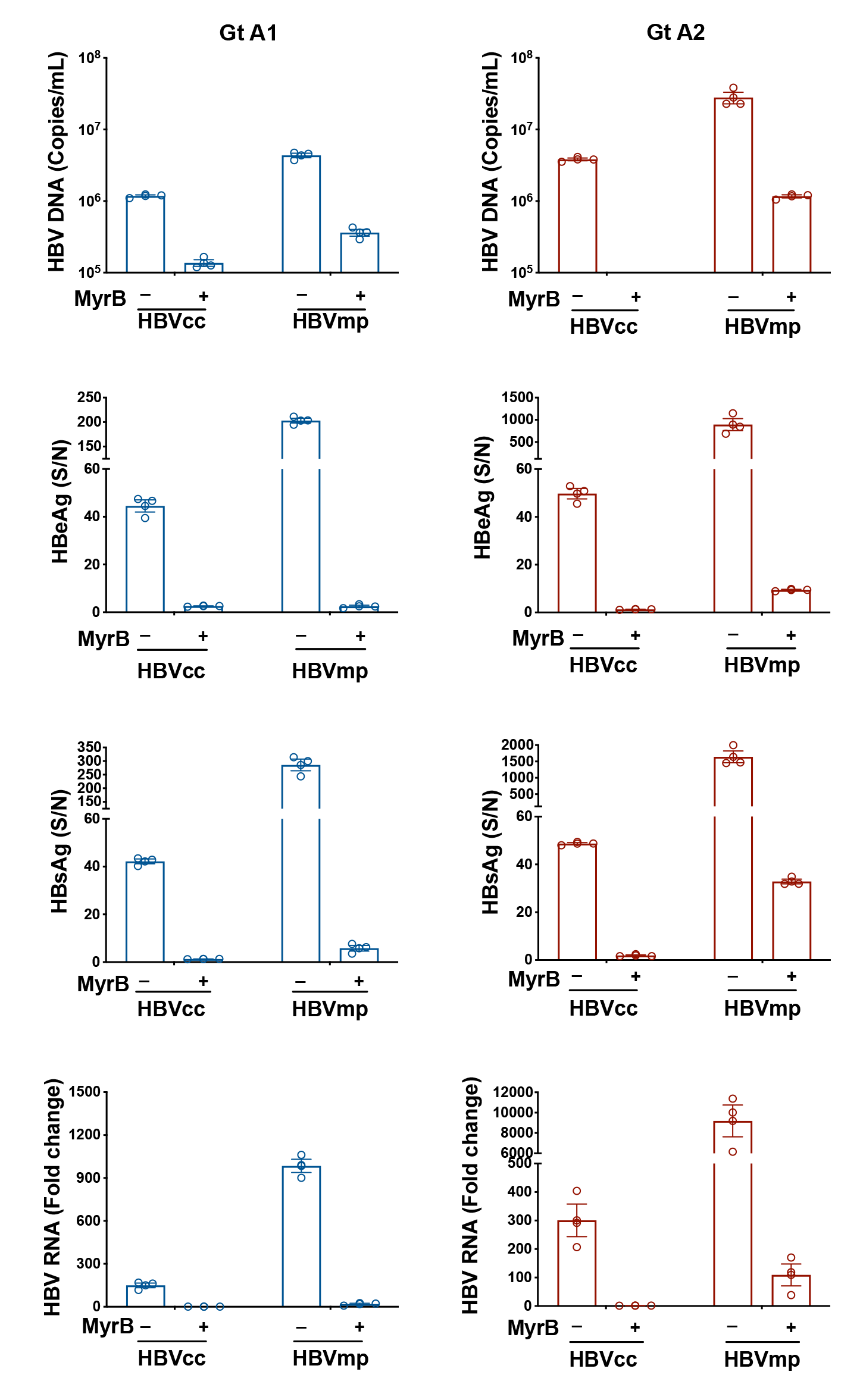

Supplement: S3 Fig — PXB-cells were infected with HBVcc and HBVmp (harvested from each infected mouse) at an MOI of 10. Myrcludex B (MyrB) treatment was used as negative control. Culture media on day 14 post-infection were harvested and assayed for HBV DNA, HBeAg and HBsAg. PXB-cells were harvested for determination of intracellular HBV RNA. Error bars indicate mean ± SEM of triplicates. (TIF) [file ppat.1012803.s003.tif]

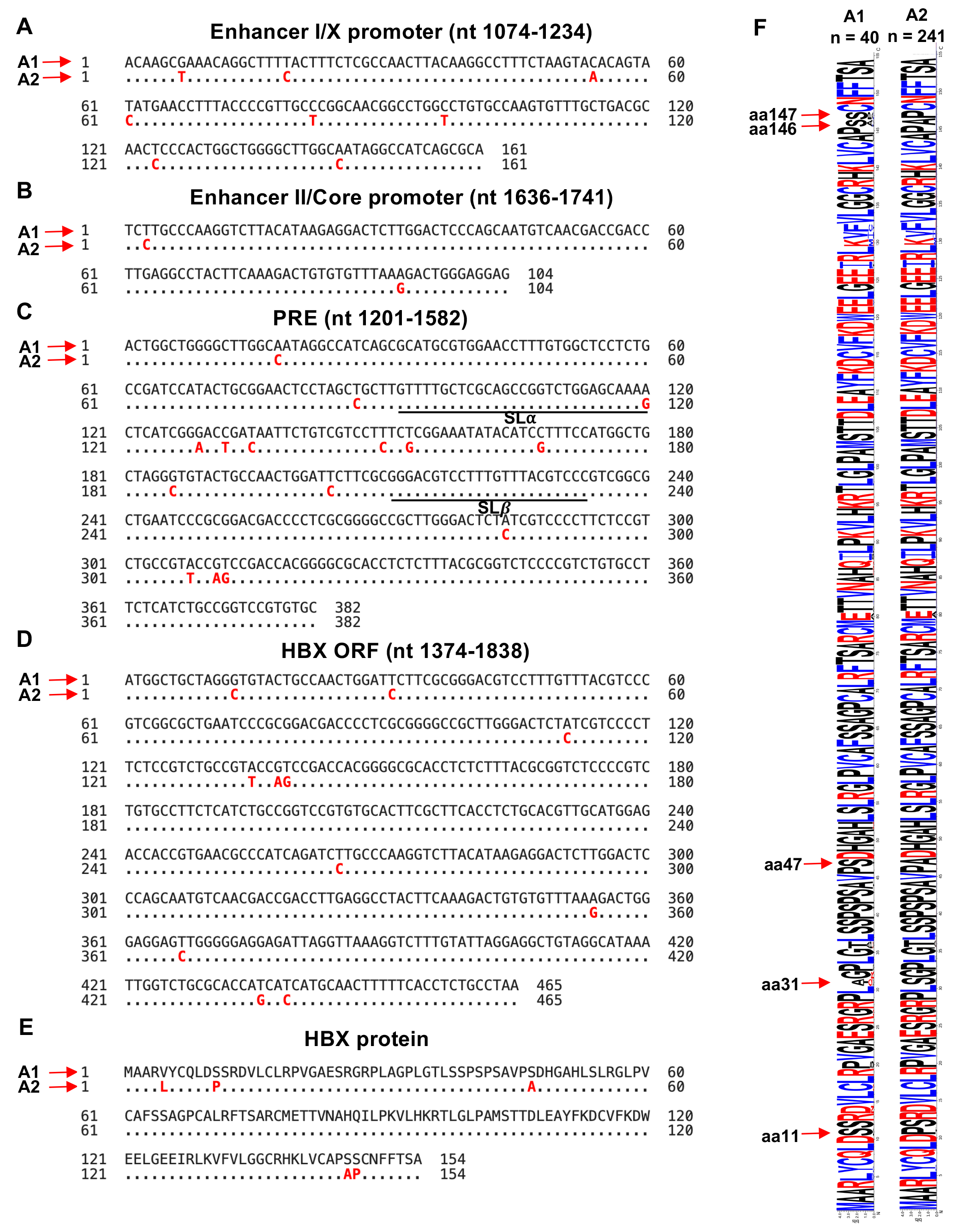

Supplement: S4 Fig — A-E, The sequence of Enhancer I/X promoter, Enhancer II/Core promoter, PRE, HBX ORF and HBx protein were compared between subtypes A1 and A2. The different DNA bases/amino acids were highlighted with red. F, Weblogo sequence alignment of HBx between all A1 and A2 sequences. The sequences of the HBx of all available HBV subtypes A1 and A2 were obtained from the NCBI database. Generated alignments and sequence conservation were visualized and calculated with the Mafft Web Service in Jalview 2.11.2.7 and Berkeley Weblogo 3 programs. The value n represents the number of HBV sequences of each genotype included in the alignment. In the alignment figure, hydrophilic amino acids were marked red, neutral amino acids black, and hydrophobic amino acids blue. Major different amino acids were indicated with red arrows above the alignment. (TIF) [file ppat.1012803.s004.tif]
