# Supplementary material for: Hepatitis B virus genotypes A1 and A2 have distinct replication phenotypes due to polymorphisms in the HBx gene
Source: PLoS Pathog. 2025 Jan 9;21(1):e1012803. doi: 10.1371/journal.ppat.1012803 (PMC11717313; doi:10.1371/journal.ppat.1012803)
Supplement: S1 Table — (DOCX) [file ppat.1012803.s005.docx]

**Supplementary Table 1.**

**Primers for HBx mutation**

| **Primers** | **Sequence (5’-3’)** |
| --- | --- |
| V5L-s: | ATGGCTGCTAGGCTGTACTGCCAAC |
| V5L-a: | GTTGGCAGTACAGCCTAGCAGCCAT |
| S11P-s: | TGCCAACTGGATCCTTCGCGGGACG |
| S11P-a: | CGTCCCGCGAAGGATCCAGTTGGCA |
| S47A-s | TCTGCCGTACCGGCCGACCACGGGG |
| S47A-a: | CCCCGTGGTCGGCCGGTACGGCAGA |
| S146A-s: | GTCTGCGCACCAGCATCATGCAACT |
| S146A-a: | AGTTGCATGATGCTGGTGCGCAGAC |
| S147P-s: | TGCGCACCATCACCATGCAACTTTT |
| S147P-a: | AAAAGTTGCATGGTGATGGTGCGCA |
